# Discovering the complexity of the metazoan transcriptome

**DOI:** 10.1186/gb4172

**Published:** 2014-04-29

**Authors:** Matthew J Hangauer, Susan Carpenter, Michael T McManus

**Affiliations:** 1 Diabetes Center, Department of Microbiology and Immunology, University of California, San Francisco, CA 94143, USA

## Abstract

Recent studies harnessing deep RNA sequencing coupled with other complementary data have revealed the complex nature of metazoan transcriptomes.

## The deeper you look, the more you find: pervasive transcription

The advent of deep sequencing technology has ushered in a new era of genomics research. The application of deep sequencing to RNA analysis has shown that the majority of the genomic sequence is transcribed in humans and other metazoan species [1]. These analyses have resulted in the identification of thousands of novel coding and non-coding genes. Yet, it is clear that the full catalog of transcription remains elusive. For example, when presumed non-transcribed human intergenic sequence was studied in great depth, a complex web of overlapping, spliced transcripts of very low abundance was found [2]. These and other findings indicate that the complexity of the transcriptome is currently underappreciated.

In 2012, as part of the ENCODE project, which focused initially on humans, Djebali *et al. *[1] showed the staggering complexity and expanse of the human transcriptome. At least three-quarters of the genome produces transcripts, many of which demonstrate complex splicing and specific expression patterns. Recent results from the modENCODE project by Brown *et al. *[3] analyzing the *Drosophila* transcriptome have shown similarly impressive complexity and some surprising differences compared with humans.

## Digging even deeper: splicing

The ENCODE project team observed that human genes do not follow a minimalistic isoform expression strategy; instead each gene simultaneously produces up to 10 to 12 isoforms [1]. In contrast, Brown *et al. *[3] observed that in *Drosophila*, most genes have relatively few isoforms. In fact, 42% of *Drosophila* genes encode only a single transcript isoform, whereas in mammals, 95% of genes have multiple expressed isoforms [1,4]. This difference in number of isoforms per gene is unlikely to be due to uneven sampling or read depth between humans and *Drosophila.* The *Drosophila* data consist of over 12 billion read pairs from many tissues and conditions, with enough read depth and conditions to identify isoforms of most genes, and are at least on a par with the read depth of the human data. Interestingly, in *Drosophila*, the majority of alternatively spliced genes encode only a single protein sequence and vary only in the first exon, through either alternative promoter usage or splicing [3]. Genome-wide analysis of human isoform protein-coding capacity has not been reported, and it will be interesting to determine whether human alternative splicing significantly affects the protein sequence.

Splicing in *Drosophila* was found to be highly tissue-specific, with over 50% of splicing events changing significantly between tissues [3]. Tissue-specific splicing appears to be more prevalent than sex-specific or developmental stage-specific splicing. In fact, sex-specific splicing was only found in sex tissues present exclusively in males or females. The underlying reasons for this dominance of tissue-specific splicing in *Drosophila* are unclear, but it may be due to a variety of causes, including the strongly tissue-specific expression or regulation of different components of splicing machinery.

Though Brown *et al. *[3] found that *Drosophila* genes have fewer isoforms than human genes, in general, they observed very complex splicing of some *Drosophila* genes. Specifically, they found that 47 genes each have an extremely high number (more than 1,000) of splice variants and are expressed primarily in developing and adult neural *Drosophila* tissue. These transcripts are strongly enriched for RNA-editing, possessing 3′ UTR extensions, and the total count of these genes’ isoforms makes up half of all transcripts in *Drosophila*. The biological significance of the extreme splicing of these genes is unclear, and it is currently unknown whether orthologs of these genes have similar splicing patterns in other organisms.

## Venturing into the dark matter of the genome

Utilizing extensive RNA-seq data, Brown *et al. *[3] observed that, similar to humans, both antisense and intergenic non-coding transcription is prevalent in *Drosophila*. In particular, *Drosophila* gonad tissue was found to express many previously unknown transcripts, consistent with observations in testes from other species [5,6]. Indeed, it has been hypothesized that testes tissue has a permissive chromatin environment allowing transcription, perhaps serving as a gene creation tissue during evolution [6].

Most antisense transcription in *Drosophila* results from overlapping mRNAs on the opposite strand and the majority is the result of overlapping UTRs. However, 21% of *Drosophila* long noncoding RNAs (lncRNAs) are antisense to mRNAs (compared with 15% for humans) and some lncRNAs overlap multiple mRNAs forming ‘sense-antisense gene-chains’, similar to those seen in mammals [7]. The degree to which sense and antisense transcripts are present in the same cells, compared with mutually exclusive expression within distinct cells in a cell population, is unclear and is an important avenue for future research.

Earlier studies in *Drosophila* have catalogued lncRNAs using non-stranded RNA-seq data of limited read depth and tissue breadth. Using a vast set of stranded RNA-seq data, Brown *et al. *[3] found up to 4,000 candidate lncRNA genes with no predicted open reading frame (ORF) longer than 100 amino acids. Further refinement of this catalog, including removal of putative conserved short protein-coding genes, resulted in a catalog of about 2,000 lncRNAs. In comparison, there are 13,870 human lncRNA genes annotated in GENCODE v19. Due to the low expression of most lncRNAs and poor read coverage, Brown *et al.* catalogued the full structures of only a few hundred *Drosophila* lncRNAs. In comparison, human lncRNAs discovered from RNA-seq and other complementary deep sequencing data also suffer from incomplete structures [5,8]. As a result, it is unclear whether the different total lncRNA count in humans and *Drosophila* reflects actual lncRNA numbers or is an artifact of incomplete annotation. In addition, Brown *et al.* only studied polyadenylated RNA, so it is possible that there are many nonpolyadenylated lncRNAs that remain unidentified: the overall complexity of the *Drosophila* non-coding transcriptome could currently be underestimated.

Putative lncRNAs that have not been experimentally evaluated for protein-coding capacity could be novel protein-coding genes. High throughput refinement of candidate lncRNAs to remove very short peptide-encoding genes, such as the *Drosophila* gene tarsel-less (11 amino acids), is currently not technically feasible. Ribosomal profiling data, if available across all tissues of interest, could be used to identify only those transcripts that have no physical association with the ribosome. However, recent studies have shown that lncRNAs can associate with the ribosome yet not encode a protein [9]. Furthermore, mass spectrometry has been used effectively to identify some small peptide-encoding genes [10]. Unfortunately, mass spectrometry is prone to false negatives and therefore is not a reliable method to identify all short peptide-encoding genes exhaustively, a requirement if it were to be used as a robust filter for lncRNAs. As a result, there is currently no high throughput empirical or computational approach to definitively segregate non-coding from coding transcripts with absolute confidence. This is a major challenge for the lncRNA field and new approaches are needed.

Despite the incompleteness of lncRNA annotations, it is nonetheless clear that lncRNAs are frequently present within non-coding regions of the genome known to be functional. lncRNAs are strongly enriched for trait-associated single nucleotide polymorphisms (SNPs) in humans [8]. In *Drosophila*, of all the novel coding and noncoding genes Brown *et al.* discovered, only lncRNAs overlap previously molecularly defined mutations with phenotypes [3]. This evidence, as well as a growing list of examples of functional human lncRNAs containing trait-associated SNPs (such as MIAT), has supported the possibility that much of the intergenic functional sequence in organisms acts through non-coding RNA rather than, or in addition to, DNA. However, it remains to be experimentally determined what fraction of, and which, lncRNAs act as functional transcripts.

## Where are we going and how do we get there?

**Figure 1:**
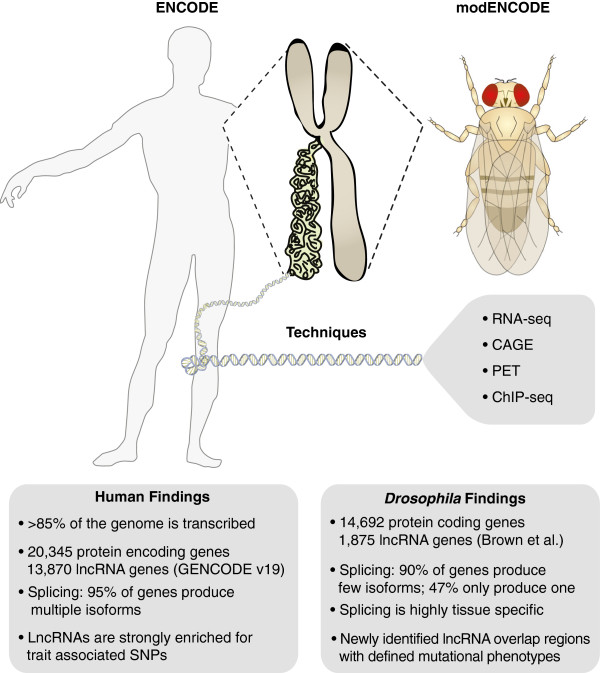
**Discovering the complexities of human and *Drosophila *transcriptomes.** The ENCODE (left-hand side) and modENCODE (right-hand side) projects have used a diverse set of techniques to provide an unprecedented view of the human and *Drosophila* transcriptomes.

Deep sequencing technology has allowed for an unprecedented view of transcriptomes, revealing tremendous complexity in transcript identity, splicing and expression patterns. Complementary techniques, including RNA-seq, CAGE, 5′ and 3′ end sequencing and others have provided large amounts of detailed information. The recent studies described here from the ENCODE and modENCODE projects exemplify what can be learned from the application of deep sequencing approaches to transcriptomes (Figure 1).

Despite this impressive array of technologies and large-scale collaborative efforts to apply them to transcriptomes, a fully comprehensive catalog of any organism’s transcriptome remains elusive. Indeed, even the complete saturation of sampling of all RNA species within a single cell type has yet to be accomplished. Many technical hurdles remain. First and foremost, what read depth is required to saturate sampling of all transcripts? The discovery of very low abundance transcripts illustrates how difficult it may be to answer this question [2]. Can a transcript with apparent expression in 1 out of 10 cells be functional? 1 out of 100? 1 out of 1,000? Is there meaningful biological information in many or few of these low abundance transcripts? Determining the limits of transcript abundance for functionality will be important in directing future efforts toward a full understanding of the transcriptome.

There are many additional technical challenges on the horizon. RNA transcripts may be capped or not, polyadenylated or not, vary drastically in size from a few nucleotides to megabases, and overlap each other in complex ways. Current deep sequencing technology does not allow for very long read lengths and extremely deep read depths at an affordable cost, yet this may be the only approach to unequivocally define full transcript structures, particularly of lowly expressed transcripts in complex loci. Another challenge on the horizon is the analysis of single cell transcriptomes. Due to difficulties in reliably isolating single cell RNA from tissues and robustly sequencing RNA from a single cell, the amount and biological importance of single cell heterogeneity of RNA expression remains controversial.

Despite these challenges, the studies by Brown *et al. *[3] and Djebali *et al. *[1] have provided important insight into the nature of the *Drosophila* and human transcriptomes. These studies and others to come will serve as fundamental resources for increasing our understanding of the transcriptome in its complicated beauty.

## Abbreviations

lncRNA: long noncoding RNA; SNP: single nucleotide polymorphism; UTR: untranslated region.

## Competing interests

The authors declare that they have no competing interests.
